# Improving cataract outcomes through good postoperative care

**Published:** 2016

**Authors:** Nick Astbury

**Affiliations:** Clinical Senior Lecturer: International Centre for Eye Health, London School of Hygiene and Tropical Medicine, London, UK.

**Figure:**
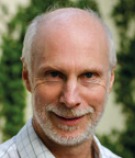
Nick Astbury

Cataract surgery is one of the most successful and frequently performed operations worldwide, and yet cataract remains the commonest cause of global blindness.^1^ This is in part due to the shortage and uneven distribution of trained personnel in some countries. More worryingly, a high rate of cataract blindness also reflects poor visual outcomes after surgery, as has been documented in many RAAB (rapid assessment of avoidable blindness) studies.^2^ In turn, poor visual acuity outcomes can be the result of inadequate pre-operative assessment (such as inaccurate biometry and/or a failure to detect signs which indicate that surgery may be complicated), complications during the surgical procedure itself, and poor postoperative management (including a lack of refraction).

Postoperative care does not always receive the attention it deserves. For example, when looking for information online, there are six times as many search results available about cataract surgery as there are about postoperative care -despite the latter being a vital component in achieving a good visual outcome.

**ABOUT THIS ISSUE**

**Figure:**
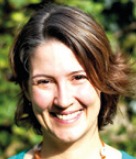
Elmien Wolvaardt Ellison

Editor: *Community Eye Health Journal*, International Centre for Eye Health, London, UK.

Responsibility for our patients does not end when they leave the operating theatre – ensuring good eye health and visual outcomes in the long term also requires good postoperative care, counselling and follow-up. This issue offers practical advice and emphasises the importance of involving patients and family members in postoperative care.

**Figure:**
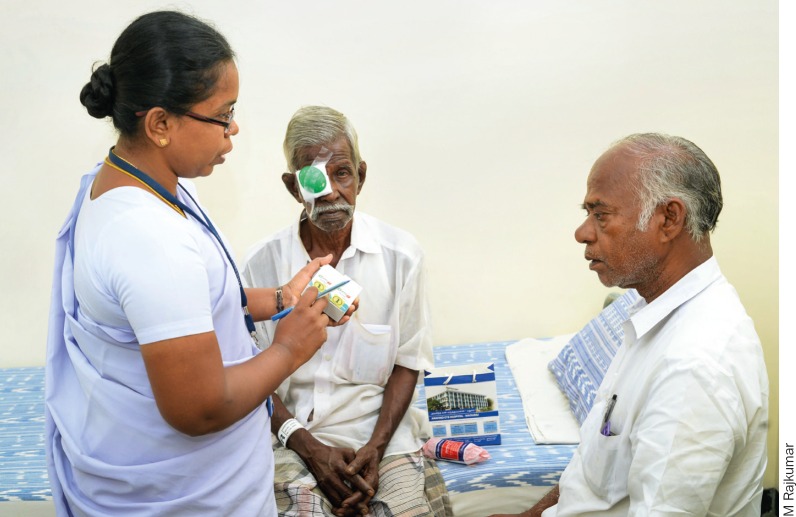
A nurse explains how to apply postoperative medication. INDIA

In this issue of the *Community Eye Health Journal*, Dr George Ohito from St Mary's Mission Hospital, Langata, Kenya, describes postoperative care as “an integral part of cataract management, with the objectives of minimising patient discomfort and pain, preventing injury and complications, and improving surgical and vision outcomes” (page 26). This definition covers all aspects: counselling, advice following surgery, and – importantly – postoperative refraction. The latter is important as there is often residual refractive error after cataract surgery, whether from astigmatism or inaccurate biometry.

The VISION 2020 initiative^3^ requires three components-trained personnel, equipment and facilities, and community participation. Good postoperative care starts even before surgery and involves patients and the community by means of counselling to allay fears and manage expectations (see the article on page 23). Patients may not access eye care services because they fear surgery or worry that they won't be able to work after an operation. Patients and their carers need reassurance and advice and must know what to do when they return home. If this is done well, and the outcomes are good, others in the community will have confidence in the eye team and be more likely to present themselves for surgery when their time comes.

Although the surgical team's responsibility doesn't stop when the patient leaves the operating theatre, patients also have a role to play. On page 25, authors Aravind, Baam and Ravindran suggest that there should be a 50:50 partnership between the patient and the eye care team so that both parties contribute to a successful visual outcome. Patients must know how to look after their operated eye and be empowered to take immediate action if they notice any symptoms or signs that might indicate a complication. This is why good counselling – before patients leave the hospital – is so important.

**‘To achieve a good outcome from cataract surgery, a team effort is needed’**

In this edition, we cover many aspects of postoperative care, tailored for different settings. Patients may be treated as day cases or may be in-patients who live far from the hospital. The timing of postoperative refraction will vary, but the important point is that it is done. Patients also have different home circumstances, and the postoperative advice given to them must be adjusted accordingly.

To achieve a good outcome from cataract surgery, a team effort is needed – community eye care workers, nurses, counsellors, eye surgeons and optometrists, as well as the patients and their carers-all have to have an understanding of the cataract journey (from first diagnosis to discharge), the complications that may arise, and how they can be prevented or their impact minimised.

For postoperative care to be **consistently** successful, systems need to be in place to support the eye team in this important work. This can include having a checklist to ensure that every patient has been given the care and information they need before leaving the hospital, having written information ready to hand out to patients, and undertaking regular monitoring. A culture of honesty and learning from mistakes-rather than denial and blame-should also be encouraged. A beautifully completed cataract operation should only be counted a success when the patient is back home, enjoying seeing again, with appropriate correction of any refractive error.

To use a sporting analogy, the end of the operation signals half-time, but the game can still be lost if attention is not paid to the postoperative period and refraction (the second half). The game is won by a joint team approach and not just by one star player – and remember that the patient is a member ofthat team!
